# Why breed disease-resilient livestock, and how?

**DOI:** 10.1186/s12711-020-00580-4

**Published:** 2020-10-14

**Authors:** Pieter W. Knap, Andrea Doeschl-Wilson

**Affiliations:** 1 Genus-PIC, 24837 Schleswig, Germany; 2 The Roslin Institute and R(D)SVS, University of Edinburgh, Easter Bush Estate, Edinburgh, EH25 9RG Scotland, UK

## Abstract

**Background:**

Fighting and controlling epidemic and endemic diseases represents a considerable cost to livestock production. Much research is dedicated to breeding disease resilient livestock, but this is not yet a common objective in practical breeding programs. In this paper, we investigate how future breeding programs may benefit from recent research on disease resilience.

**Main body:**

We define disease resilience in terms of its component traits resistance (R: the ability of a host animal to limit within-host pathogen load (PL)) and tolerance (T: the ability of an infected host to limit the damage caused by a given PL), and model the host's production performance as a reaction norm on PL, depending on R and T. Based on this, we derive equations for the economic values of resilience and its component traits. A case study on porcine respiratory and reproductive syndrome (PRRS) in pigs illustrates that the economic value of increasing production in infectious conditions through selection for R and T can be more than three times higher than by selection for production in disease-free conditions. Although this reaction norm model of resilience is helpful for quantifying its relationship to its component traits, its parameters are difficult and expensive to quantify. We consider the consequences of ignoring R and T in breeding programs that measure resilience as production in infectious conditions with unknown PL—particularly, the risk that the genetic correlation between R and T is unfavourable (antagonistic) and that a trade-off between them neutralizes the resilience improvement. We describe four approaches to avoid such antagonisms: (1) by producing sufficient PL records to estimate this correlation and check for antagonisms—if found, continue routine PL recording, and if not found, shift to cheaper proxies for PL; (2) by selection on quantitative trait loci (QTL) known to influence both R and T in favourable ways; (3) by rapidly modifying towards near-complete resistance or tolerance, (4) by re-defining resilience as the animal's capacity to resist (or recover from) the perturbation caused by an infection, measured as temporal deviations of production traits in within-host longitudinal data series.

**Conclusions:**

All four alternatives offer promising options for genetic improvement of disease resilience, and most rely on technological and methodological developments and innovation in automated data generation.

## Background

Worldwide, infectious diseases reduce production performance, fertility, and survival of livestock, and therefore form a limiting factor to the sustainability and profitability of livestock production and to carbon neutral farming, which has become a major goal in many countries. Focusing on the profitability element, Table 1 summarizes the estimated costs of fighting disease on the national level, compared to the value of the estimated genetic trend in production and/or reproduction traits around the reporting year.

**Table 1:** Cost of fighting disease versus the annual value of genetic improvement ($\mathrm{\Delta G}$) in (re)production traits

Area	Year	Disease	Total cost (M€/year)	Cost per head	$\mathrm{\Delta G}$ per head	Cost/$\mathrm{\Delta G}$
NLD	1997	CSF	2340	153		
TWN	1997	FMD	1415	119		
GBR	2001	FMD	12,864	306		
KOR	2010	FMD	1401	87		
CHN	2019	ASF	22,768	96		
AUS	2006	PAR	389	3.7	0.49	7.7
NLD	2007	BLT	170	37.3	14	2.7
ENG	2010	BTB	127	23.2	4.4	5.2
CAN	2010	PRRS	95	4.5	0.88	5.1
USA	2013	PRRS	860	7.7	1.7	4.6
EUR	2013	PRRS	1660	6.6	1.6	4.1

Areas: *NLD* Netherlands; *TWN* Taiwan; *GBR* Great Britain; *KOR* South Korea; *CHN* China; *AUS* Australia; *ENG* England; C*AN* Canada; *EUR* Austria, Belgium, Denmark, France, Germany, Italy, Netherlands, Poland, Russia, Spain and United Kingdom

Diseases: *CSF* classical swine fever; *FMD* foot and mouth disease (ungulates); *ASF* African swine fever; *PAR* ectoparasites (sheep); *BLT* bluetongue (ungulates); *BTB* bovine tuberculosis; *PRRS* porcine respiratory and reproductive syndrome

More details, including references and footnotes, are in Additional file 1: Table S1

The first five entries in Table 1 represent cases where a major epidemic was dealt with by population-wide culling, which leads to costs of the order of € 100 to € 200 per animal, dwarfing any achievable earnings from genetic improvement (note that the cost of fighting African swine fever in China includes only the material loss and not the resulting 2.5-fold increase in the retail price of pig meat). The next six entries represent the ongoing annual costs of endemic disease control (and of one minor epidemic) and show that those costs range from 2.7 to 7.6 times the annual value of genetic improvement ($\mathrm{\Delta G}$) for production and/or reproduction traits. Similar information can be found in Chapters 16–20 in [1].

In line with the above, and considering antimicrobial resistance and changes in farming practices that heighten disease emergence, disease resilience has become one of the most desirable attributes of livestock. Nevertheless, to date, the livestock breeding sector practices little explicit selection for traits related to disease resilience. Several pig and poultry breeding companies select for reduced mortality rates (which also have high economic values, e.g. [2] for pigs; [3] for turkeys) based on data recorded on close relatives of nucleus selection candidates, grown in commercial conditions. Although this can lead to solid $\mathrm{\Delta G}$ trends in survival and production (e.g. Fig. 3 in [4]), overall mortality has many more causes than infectious disease—so the relationship to disease resilience is unclear and variable, and it is difficult to extrapolate to newly emerging diseases. Other companies aim at breeding for increased host resistance to specific diseases (e.g. [5] in pigs) which usually requires routine challenge tests (e.g. [6, 7] in salmon; and [8] in poultry) or extensive recording of resistance traits in natural challenge conditions (e.g. [9] in sheep; [10] in cattle; and [11] in rabbits).

Recently disease tolerance has been proposed as an alternative breeding goal trait [12–14], but to our knowledge, to date no breeding company carries out explicit selection for increased tolerance of animals to any type of infection.

The terminology around robustness, disease resilience, resistance, and tolerance is confusing: the latter three terms are often used interchangeably and quantified by the same phenotype (e.g. mortality), making explicit focus on, e.g., resistance and tolerance impossible. Some recent studies have defined resistance and tolerance as component traits of disease resilience [14–16]. These studies indicate that explicit selection for resistance and tolerance would require extensive routine data recording, which often can only be obtained in disease challenge tests. This would lead to considerable investment for breeding companies, and the associated cost–benefit analysis requires sound estimates of the economic values of these traits. However, the required theoretical framework to determine (i) the relative contributions of resistance and tolerance to genetic improvement of disease resilience and (ii) the associated economic values is currently lacking.

This paper has three objectives. First, to develop a unified framework to define resilience in terms of its component traits resistance and tolerance. Second, to derive the economic values of these three traits, with an example for PRRS in pigs. Third, to discuss applications and the way forward in breeding disease resilient livestock.

## Main text

### Theoretical framework: disease resilience and its component traits resistance and tolerance

Disease resilience in the context of livestock production was first defined as the ability of a host animal to maintain a reasonable level of productivity when challenged by infection [17]. Disease resilience is assessed by comparing the production performance of an individual or a family (e.g. a sire's daughter group) between environments with different levels of pathogen load ($\mathrm{PL}$) [18]. In more quantitative terms, disease resilience can be defined as the reaction norm of performance on environmental $\mathrm{PL}$, i.e. as a continuous trait [19]. Disease resilience captures two complementary host defence mechanisms against pathogens: resistance and tolerance [20].

Disease resistance is the ability of a host animal to limit its within-host pathogen load, either by preventing infection in the first place or by inhibiting within-host pathogen replication [21, 22]. In simple terms: how does the host and its immune system respond to the environmental $\mathrm{PL}$? As such, it determines to what extent the environmental PL leads to within-host PL; thus, it is most accurately quantified by continuous measures of within-host PL (e.g. viremia or bacterial / parasite counts). Low within-host PL corresponds to high resistance.

Disease tolerance is the ability of an infected host to limit the damage caused by a given within-host $\mathrm{PL}$ [21, 22] without necessarily reducing this $\mathrm{PL}$ as such [23]. In simple terms: how does the body cope with preventing or repairing the damage inflicted either by the pathogen or by the activated immune system? In quantitative livestock terms: the change in host performance as within-host $\mathrm{PL}$ changes, i.e. the slope of the reaction norm of production performance on within-host $\mathrm{PL}$ [24]: a continuous trait again. Confusingly, the term "tolerance" has often been used to refer to the resilience mechanism that is based on environmental $\mathrm{PL}$ as described above [25–27].

Robustness, in the context of intensive livestock production, refers to the combination of a high production potential with high resilience to external stressors (such as environmental PL), allowing for unproblematic expression of that production potential in a wide variety of environmental conditions [2, 28, 29]. Thus, robustness is very similar to general resilience to a variety of stressors, focusing in particular on high-performance genotypes.

**Fig. 1:**
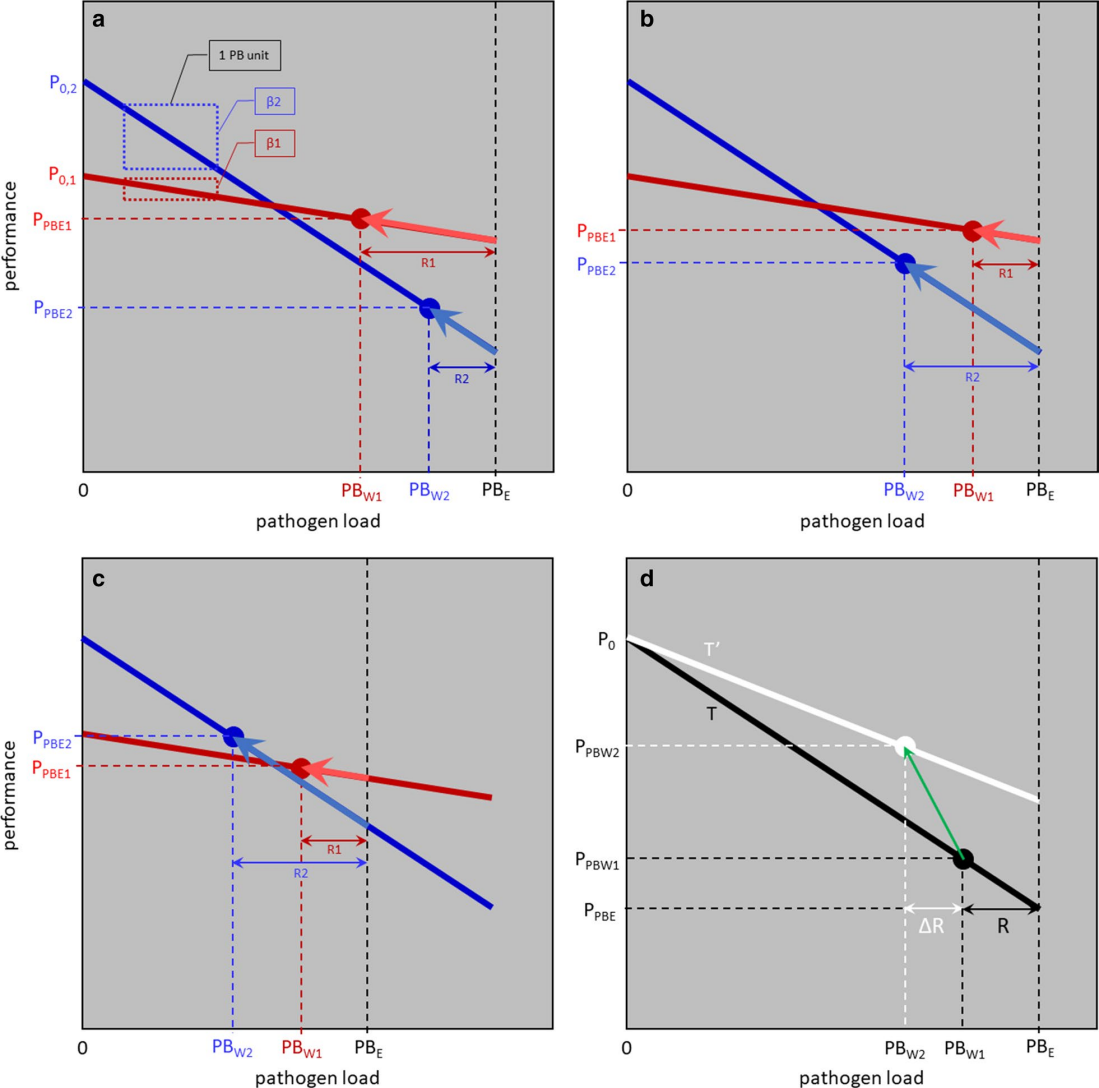
Reaction norm models for disease resilience. **a–c** A model of realized performance in infectious conditions (${\mathrm{P}}_{\mathrm{PLE}}$) as it depends on environmental pathogen load (${\mathrm{PL}}_{\mathrm{E}}$), host performance potential (${\mathrm{P}}_{0}$), host resistance ($\mathrm{R}$) and host tolerance ($\mathrm{T}=-1/\upbeta$; with the slope of the regression of performance versus $\mathrm{PL}$, $\upbeta \le 0$) for two host animals with different levels of ${\mathrm{P}}_{0}$, R and T, exposed to different ${\mathrm{PL}}_{\mathrm{E}}$ levels. $\mathrm{T}$ and $\mathrm{R}$ are favourably correlated in (**a**) and unfavourably in (**b**) and (**c**); ${\mathrm{PL}}_{\mathrm{E}}$ is lower in (**c**) than in (**a**) and (**b**). Resistance reduces ${\mathrm{PL}}_{\mathrm{E}}$ to within-host pathogen load (${\mathrm{PL}}_{\mathrm{W}}$) with performance recapture along the reaction norm to the ${\mathrm{P}}_{\mathrm{PLE}}$ level. In (**a**) ${\mathrm{P}}_{\mathrm{0,2}}>{\mathrm{P}}_{\mathrm{0,1}}$, but ${\mathrm{P}}_{\mathrm{PLE},2}<{\mathrm{P}}_{\mathrm{PLE},1}$ because individual 2 is less resistant to infection (lower reduction from ${\mathrm{PL}}_{\mathrm{E}}$ to ${\mathrm{PL}}_{\mathrm{W}}$: ${\mathrm{R}}_{2}<{\mathrm{R}}_{1}$) and also less tolerant to it (steeper slope: ${\upbeta }_{2}$ is more strongly negative than ${\upbeta }_{1}$). In (**b**), the $\mathrm{T}$ levels are the same as in (**a**), but ${\mathrm{R}}_{2}>{\mathrm{R}}_{1}$; this causes a stronger reduction from ${\mathrm{PL}}_{\mathrm{E}}$ to ${\mathrm{PL}}_{\mathrm{W}}$ in individual 2, climbing a longer stretch of the reaction norm, and this reduces the ${\mathrm{P}}_{\mathrm{PLE}}$ difference. In (**c**), $\mathrm{T}$ and $\mathrm{R}$ are the same as in (**b**), but ${\mathrm{PL}}_{\mathrm{E}}$ is lower; hence individual 2′s stronger $\mathrm{R}$ can now reduce ${\mathrm{PL}}_{\mathrm{E}}$ to a more favourable ${\mathrm{PL}}_{\mathrm{W}}$ level, neutralizing its lower $\mathrm{T}$; with that its ${\mathrm{P}}_{\mathrm{PLE}}$ becomes higher. (**d**) A model of improving resilience through increases in $\mathrm{R}$ and $\mathrm{T}$ while keeping ${\mathrm{P}}_{0}$ unchanged, see the "Economic values: theory" section below. The starting position (black dot) is based on initial resistance and tolerance levels $\mathrm{R}$ and $\mathrm{T}$, with pathogen load ${\mathrm{PL}}_{\mathrm{W}1}$ and performance ${\mathrm{P}}_{\mathrm{PLW}1}$. From there, resistance is increased by $\mathrm{\Delta R}$ and tolerance from $\mathrm{T}$ by $\mathrm{\Delta T}$ to $\text{T}^{\prime}$ (a move to a shallower reaction norm), leading to a new position following the green arrow, with performance ${\mathrm{P}}_{\mathrm{PLW}2}$ (white dot)

Figure 1a–c illustrates the quantitative relationship between the various resilience traits for two host animals (red 1 and blue 2) with different performance potential (${\mathrm{P}}_{0}$, expressed at zero $\mathrm{PL}$), different resistance levels against the pathogen ($\mathrm{R}$), and different tolerance levels to infection ($\mathrm{T}$) with environmental $\mathrm{PL}$ or challenge dose ${\mathrm{PL}}_{\mathrm{E}}$. For ease of quantification, ${\mathrm{PL}}_{\mathrm{E}}$ is specified here in within-host $\mathrm{PL}$ units corresponding to a hypothetical reference host with zero resistance to the pathogen. Similar to ${\mathrm{P}}_{0}$ and to the actual environmental $\mathrm{PL}$ in field conditions, this reference value is usually not known but is useful for quantifying the relative role of the environmental $\mathrm{PL}$ for resilience, and the economic value of resilience traits (see the "Economic values: theory" section below). Figure 1 shows resilience and tolerance, following [19], as the classical reaction norm of change in performance ($\mathrm{P}$) in relation to $\mathrm{PL}$: $\mathrm{P}={\mathrm{P}}_{0}+\upbeta \times \mathrm{PL}$ (where slope $\upbeta \le 0$ and PL refers to environmental and within-host pathogen load for resilience and tolerance, respectively).

In order to keep this model easily traceable, here we quantify tolerance as $\mathrm{T}= -1/\upbeta$, so that $\mathrm{T}\ge 0$, and a value of 0 represents complete absence of tolerance. Likewise for resistance, $\mathrm{R}\ge 0$ and a value of 0 represents complete absence of resistance. For both tolerance and resistance, a numeric increase represents improvement for the host, and a negative correlation between them is unfavourable. Resistance is measured in terms of within-host $\mathrm{PL}$ units: different host resistance levels cause a reduction of ${\mathrm{PL}}_{\mathrm{E}}$ down to different levels of the realized within-host pathogen load ${\mathrm{PL}}_{\mathrm{W}}$.

Figure 1a–c demonstrates how different levels of performance can be achieved, depending on the environmental challenge, and on an individual’s performance potential, resistance, and tolerance, and the correlations between these. These concepts are formulated in a mathematical model in Eq. (1a), where each host's actual performance at a given ${\mathrm{PL}}_{\mathrm{E}}$ level (and at this host's associated ${\mathrm{PL}}_{\mathrm{W}}$ level) is given as:

1a $${\mathrm{P}}_{\mathrm{PLE}}= {\mathrm{P}}_{0}-1/\mathrm{T}\times {\mathrm{PL}}_{\mathrm{W}}={\mathrm{P}}_{0}-1/\mathrm{T}\times ({\mathrm{PL}}_{\mathrm{E}}-\mathrm{R}).$$

Note that this can be rearranged as:

1b $${\mathrm{P}}_{\mathrm{PLE}}=\left[{\mathrm{P}}_{0}+\mathrm{R}/\mathrm{T}\right]-1/\mathrm{T}\times {\mathrm{PL}}_{\mathrm{E}},$$

i.e. a reaction norm on the environmental $\mathrm{PL}$, in line with the classical definition of resilience given above. According to Eqs. 1a and 1b, infectious challenge (${\mathrm{PL}}_{\mathrm{E}}\ge 0$) reduces performance, and resilience to the pathogen constrains this reduction: a smaller reduction for more tolerant hosts (with larger $\mathrm{T}$ in Eq. (1a)) and for more resistant hosts (with larger $\mathrm{R}$ in Eq. (1a)).

### Economic value of disease resilience traits

#### Theory

Economic values of resistance, tolerance and resilience can be derived from the relationships in Fig. 1 and Eq. (1a), as follows.

The partial derivative of Eq. (1a) with respect to resistance is:

2a $$\frac{\partial {\mathrm{P}}_{\mathrm{PLE}}}{\partial \mathrm{R}}= 1/\mathrm{T}.$$

From that, the marginal economic value (MEV) of resistance is:

2b $$\mathrm{MEV}(\mathrm{R})=\frac{\partial {\mathrm{P}}_{\mathrm{PLE}}}{\partial \mathrm{R}}\times \mathrm{MEV}\left(\mathrm{P}\right)=1/\mathrm{T}\times \mathrm{MEV}\left(\mathrm{P}\right),$$

where $\mathrm{MEV}\left(\mathrm{P}\right)$ is the MEV of the production trait under consideration. Hence improvement of resistance is worth more at lower tolerance levels (low $\mathrm{T}$) and when production performance is worth more.

Likewise, the partial derivative of Eq. (1a) with respect to tolerance is:

3a $$\frac{\partial {\mathrm{P}}_{\mathrm{PLE}}}{\partial \mathrm{T}}=\frac{{\mathrm{PL}}_{\mathrm{E}} -\mathrm{ R}}{{\mathrm{T}}^{2}},$$

i.e. a function of the environmental PL and of the population means for resistance and tolerance. This results in the following MEV for tolerance:

3b $$\mathrm{MEV}(\mathrm{T})=\frac{\partial {\mathrm{P}}_{\mathrm{PLE}}}{\partial \mathrm{T}}\times \mathrm{MEV}(\mathrm{P})=\frac{{\mathrm{PL}}_{\mathrm{E}} -\mathrm{ R}}{{\mathrm{T}}^{2}}\times \mathrm{MEV}(\mathrm{P}).$$

Hence an improvement of tolerance is worth more (i) if the infectious challenge is high (high ${\mathrm{PL}}_{\mathrm{E}}$), (ii) at lower resistance levels (low $\mathrm{R}$), (iii) at lower tolerance levels (low $\mathrm{T}$), and (iv) when production performance is worth more. When resistance is strong enough to eliminate the pathogen completely (i.e. when $\mathrm{R}\ge {\mathrm{PL}}_{\mathrm{E}}$), improvement of tolerance has a zero value. Likewise, because of the squared term ${\mathrm{T}}^{2}$ in its denominator, the economic value of tolerance increases at a diminishing rate of return with increasing levels of tolerance.

The economic value of resilience is the value of the amount of performance that is recaptured by a unit improvement in resilience, along a reaction norm from ${\mathrm{P}}_{\mathrm{PLE}}$ upwards towards ${\mathrm{P}}_{0}$. This can result from improvements in resistance and/or tolerance, in any feasible combination. Figure 1d illustrates the principle. The starting position is based on initial resistance level $\mathrm{R}$ and initial tolerance level $\mathrm{T}$, with pathogen load ${\mathrm{PL}}_{\mathrm{W}1}$ and performance ${\mathrm{P}}_{\mathrm{PLW}1}$. From there, tolerance is increased by $\mathrm{\Delta T}$ (from $\mathrm{T}$ to $\text{T}^{\prime}$) and resistance by $\mathrm{\Delta R}$, leading to a new position on a new reaction norm, with performance ${\mathrm{P}}_{\mathrm{PLW}2}$.

The increase in performance is $\Delta \mathrm{P}$:

4a $$\Delta \mathrm{P}={\mathrm{P}}_{\mathrm{PLW}2}-{\mathrm{P}}_{\mathrm{PLW}1}=\left[{\mathrm{P}}_{0}-\frac{{\mathrm{PL}}_{\mathrm{E}}-\left(\mathrm{R}+\Delta \mathrm{R}\right)}{\mathrm{T}+\Delta \mathrm{T}}\right]-\left[{\mathrm{P}}_{0}-\frac{{\mathrm{PL}}_{\mathrm{E}}-\mathrm{R}}{\mathrm{T}}\right]=\frac{{\mathrm{PL}}_{\mathrm{E}}-\mathrm{R}}{\mathrm{T}}-\frac{{\mathrm{PL}}_{\mathrm{E}}-\left(\mathrm{R}+\Delta \mathrm{R}\right)}{\mathrm{T}+\Delta \mathrm{T}}$$

Recalling Eqs. (2b) and (3b), deriving from them that $\frac{{\mathrm{PL}}_{\mathrm{E}}-\mathrm{R}}{\mathrm{T}}=\frac{\mathrm{MEV}\left(\mathrm{T}\right)}{\mathrm{MEV}\left(\mathrm{R}\right)}$, and changing to differentials (from $\Delta \mathrm{P}$ to $\mathrm{dP}$), this can be rewritten as:

4b $$\mathrm{dP}=\frac{\mathrm{MEV}\left(\mathrm{T}\right)}{\mathrm{MEV}\left(\mathrm{R}\right)}-\frac{\mathrm{T}}{\mathrm{T}+\mathrm{dT}}\times \frac{\mathrm{MEV}\left(\mathrm{T}\right)}{\mathrm{MEV}\left(\mathrm{R}\right)}+\frac{\mathrm{dR}}{\mathrm{T}+\mathrm{dT}}=\frac{\mathrm{dT}}{\mathrm{T}+\mathrm{dT}}\times \frac{\mathrm{MEV}\left(\mathrm{T}\right)}{\mathrm{MEV}\left(\mathrm{R}\right)}+\frac{\mathrm{dR}}{\mathrm{T}+\mathrm{dT}}$$

With $\mathrm{dT}$ small enough for $\mathrm{T}+\mathrm{dT}\approx \mathrm{T}$, this simplifies to:

4c $$\begin{aligned} & {\text{dP}} \approx \frac{{{\text{dT}}}}{{\text{T}}} \times \frac{{{\text{MEV}}\left( {\text{T}} \right)}}{{{\text{MEV}}\left( {\text{R}} \right)}} + \frac{{{\text{dR}}}}{{\text{T}}} \\ & = {\text{dT}} \times \frac{{{\text{MEV}}\left( {\text{R}} \right)}}{{{\text{MEV}}\left( {\text{P}} \right)}} \times \frac{{{\text{MEV}}\left( {\text{T}} \right)}}{{{\text{MEV}}\left( {\text{R}} \right)}} + {\text{dR}} \times \frac{{{\text{MEV}}\left( {\text{R}} \right)}}{{{\text{MEV}}\left( {\text{P}} \right)}} = \frac{{{\text{dT}} \times {\text{MEV}}\left( {\text{T}} \right) +{\text{dR}} \times {\text{MEV}}\left( {\text{R}} \right)}}{{{\text{MEV}}\left( {\text{P}} \right)}} \\ \end{aligned}.$$

Note that for $\mathrm{dT }= 0$, $\frac{\mathrm{dP}}{\mathrm{dR}}=1/\mathrm{T}$, as in Eq. (2a); for $\mathrm{dR }= 0$, $\frac{\mathrm{dP}}{\mathrm{dT}}=\frac{{\mathrm{PL}}_{\mathrm{E}}-\mathrm{R}}{{\mathrm{T}}^{2}}$, as in Eq. (3a).

The amount of recaptured performance of Eq. (4c) represents an economic value of $\mathrm{dP}\times \mathrm{MEV}(\mathrm{P})\approx \mathrm{dT}\times \mathrm{MEV}(\mathrm{T})+\mathrm{dR}\times \mathrm{MEV}(\mathrm{R})$. Hence, the MEV of disease resilience follows from the MEV of its component traits resistance and tolerance, weighted by their specific contributions to the change in resilience. As such, the MEV of resilience cannot be quantified without knowledge of those contributions.

### Case study: the economic value of resilience of growing pigs to PRRS virus infections

PRRS is widely considered as one of the most economically important viral diseases in pigs worldwide [30], yet the economic value of resilience to this disease is currently not known. Estimates can be derived by applying the above model to data from a large scale PRRS virus challenge experiment where piglets were infected with the same dose of a virulent PRRS virus strain at about 30 days of age [31]. Lough et al. [32] derived tolerance estimates from individual body weight and viremia records on 1011 of these young pigs by linear regression of performance on within-host PL. Here, performance was the average growth rate from 14 to 42 days post-infection; pathogen load was AUC(logVL), i.e. the area under the curve of log-transformed viremia measurements, in that same period ( [32], see their Additional file 2). The average change in growth rate in this population per unit increase of viremia and the standard deviation of the 1011 individual regression coefficients were $\upbeta =-0.002660$ and ${\upsigma }_{\mathrm{P}}(\upbeta )=0.00418$ kg/d per AUC(logVL), respectively. From that, T equals 375.9.

The genetic standard deviations (${\upsigma }_{\mathrm{G}}$) of growth rate, of AUC(logVL), and of the reaction norm slope in this data were 0.0549 kg/d, 9.22 AUC(logVL) units, and 0.0000924 kg/d per AUC(logVL) unit, respectively. From the ${\upsigma }_{\mathrm{G}}$ of growth rate (i.e. of ${\mathrm{P}}_{\mathrm{PLE}}$), the ${\upsigma }_{\mathrm{G}}$ of ${\mathrm{P}}_{0}$ can be derived (see Additional file 2) to be 0.011 kg/d. For comparison, [33] and [34] published genetic standard deviation estimates for growth rate in pigs up to 30 kg body weight in high-health environments of 0.026 kg/d in Japanese Duroc and ranging from 0.014 to 0.016 kg/d in Danish Landrace, Yorkshire and Duroc pigs. These values are closer to our 0.011 estimate of ${\upsigma }_{\mathrm{G}}$ for ${\mathrm{P}}_{0}$ than to the 0.0549 estimate of ${\upsigma }_{\mathrm{G}}$ for realized performance in deliberately low health conditions, as expected. Likewise, [35] estimated ${\upsigma }_{\mathrm{G}}$ = 0.038 kg/d in Danish commercial crossbreds.

In Denmark, the MEV of growth rate in pigs of this age range was estimated as DKK 110 = € 15 per kg/d [36]. In the absence of trade-offs with resistance and tolerance, an improvement of the genetic potential for nursery-stage growth rate ${\mathrm{P}}_{0}$ by one ${\upsigma }_{\mathrm{G}}$ unit then has a value of 0.011 × 15 = € 0.165 per pig.

When challenged with PRRS virus infection, and in the absence of trade-offs with performance potential and tolerance, an improvement of PRRS resistance that would lead to a reduction of AUC(logVL) by one ${\upsigma }_{\mathrm{G}}$ (9.22 units) would increase nursery-stage growth rate (in this population with its average tolerance level, and in this environment with its ${\mathrm{PL}}_{\mathrm{E}}$) by 9.22 × 0.00266 = 0.0245 kg/d. Such an increase in resistance has a value of 0.0245 × 15 = € 0.37 per pig.

For the MEV of PRRS tolerance, consider a tolerance reaction norm that is one ${\upsigma }_{\mathrm{G}}$ (i.e. 0.0000924 kg/d per AUC(logVL) unit) shallower (such that $\upbeta$ = –0.002568 and therefore $\mathrm{T}$ = 389.5) than the current population average ($\mathrm{T}$ = 375.9). At zero PL, the two tolerance levels lead to the same growth rate, and tolerance has a zero MEV; with increasing PL_W_ viremia levels (due to decreasing resistance levels in the host), the contrast in growth rate increases and, therefore, the MEV of tolerance increases. In other words, the MEV of tolerance depends on the resistance level in the target population, as per Eq. (3b). At the highest viremia level in this data (i.e. 181.4 AUC(logVL) units), in this population with its average resistance level, and in this environment with its ${\mathrm{PL}}_{\mathrm{E}}$, an improvement of PRRS tolerance by one ${\upsigma }_{\mathrm{G}}$ would increase nursery-stage growth rate by 0.0000924 × 181.4 = 0.01676 kg/d, with an economic value of € 0.25 per pig (again in the absence of trade-offs with performance potential and resistance).

An improvement of resilience due to simultaneous improvement of resistance and tolerance (each by one ${\upsigma }_{\mathrm{G}}$, i.e. $\mathrm{\Delta R}$ = 9.22 and $\mathrm{\Delta T}$ = 13.6) would lead to a maximum (i.e. calculated at the highest viremia level in this data) performance recapture of $\mathrm{\Delta P}$ = 0.0245 + 0.0168 = 0.041 kg/d, with an economic value of € 0.37 + 0.25 = € 0.62 per pig. As an indirect selection strategy for performance at the highest viremia level in this data, it would deliver 0.62/0.165 = 3.7 times the economic value achieved by selection on ${\mathrm{P}}_{0}$.

In this example, production performance refers to nursery-stage growth rate of pigs in a disease challenge test. The same calculations could be applied to other relevant production traits of the grower-finisher pig, such as post-nursery growth rate, feed intake, or mortality, as well as to reproductive performance of sows, preferably recorded in field conditions. Given the devastating effects of PRRS on reproduction traits [37], the associated economic value for resilience as measured in terms of reproductive performance may well be higher than that presented in the example above.

In summary, our calculations support the evidence from Table 1 that the economic value of resilience is high.

### Obstacles for genetic improvement of disease resilience in practice

The above reaction norm model has proven useful for modelling the interaction between resilience component traits and for deriving economic values that can serve as the basis for cost–benefit analyses. In this section, we address several hurdles that may hinder the application of such models for actual data analysis and for genetic improvement of disease resilience in practice, followed by a section that proposes solutions.

#### Resilience component traits are difficult to measure or estimate

Reaction norm models with ambient temperature as the physically recorded independent variable have been used frequently to study heat resilience in ruminants and pigs [38–43]. Temperature recordings are easily obtained from meteorology services or from in-house recording. By contrast, the various parameters that feature in our Fig. 1 (i.e. the within-host pathogen load ${\mathrm{PL}}_{\mathrm{W}}$, the environmental pathogen load ${\mathrm{PL}}_{\mathrm{E}}$, and the performance potential ${\mathrm{P}}_{0}$) come with considerable recording challenges. In addition, the reaction norm approach itself presents statistical challenges, in particular for estimating tolerance.

${\mathrm{PL}}_{\mathrm{W}}$ requires quantification of the amount of pathogen carried by a host animal, which involves methods ranging from counting ectoparasites (e.g. [44] for millimeter-long copepods on a sedated fish), to microscope counting of endoparasite eggs in fecal samples (e.g. [45]), and to running PCR DNA or RNA assays to establish bacterial or viral load in blood or tissue samples (e.g. [46, 47]). All these methods are well established but require skilled operators and often specialized equipment, and therefore carry significant cost. For example, Thorvaldsen et al. [48] mention 15 to 80 min work for three people to count the copepods on 20 salmon (which would cost the farm at least € 1 to € 5 per animal; [see tinyurl.com/rs4tltk]); outsourced fecal egg counting is charged at € 5 to € 15 per sample in the UK, the Netherlands, and Australia [see tinyurl.com/wqs99tp, tinyurl.com/rzkronj, tinyurl.com/w7n2tu3]; a PCR assay of PRRS viremia costs € 27 to € 42 per sample in the UK, Belgium, and USA [see tinyurl.com/wqs99tp, tinyurl.com/w4ct6ud, tinyurl.com/t5vsu9q, tinyurl.com/r99p5lu]. In comparison, outsourced performance testing of pigs for growth rate and ultrasound body composition in USA costs € 3.5 per animal [see tinyurl.com/v82tyqt]. Hence, although ${\mathrm{PL}}_{\mathrm{W}}$ recording is not prohibitively difficult, it is currently costly. Recent developments in bioimaging, low-cost high-throughput metagenomic profiling, and next-generation DNA sequencing suggest that cost-effective approaches to measuring $\mathrm{PL}$ are on the horizon [49–53].

Direct measures of ${\mathrm{PL}}_{\mathrm{E}}$, in the required PL_W_ units, rarely exist. In practice, ${\mathrm{PL}}_{\mathrm{E}}$ may be approximated by some percentile of the highest observed PL_W_ values in the population experiencing the same infectious challenge, effectively shifting the reference from a host with zero resistance to the least resistant hosts in the dataset. Similar to the common approach of using the population mean performance as a proxy of environmental PL [54], this approach relies on the assumption that the sampling distribution is a valid representation of the full distribution of PL_W_ in each environment.

In controlled challenge trials with a single specific pathogen, ${\mathrm{P}}_{0}$ may be quantified as the observed performance prior to infection or, on a genetic level, from the performance of genetically related unchallenged control animals (e.g. [55]). Without such trials, genetic estimates of ${\mathrm{P}}_{0}$ may be derived from performance measures of genetically related individuals in "clean" farming environments, such as a nucleus farm in pig or poultry breeding. As these are unlikely to be entirely free of pathogens, ${\mathrm{P}}_{0}$ will typically be underestimated to an unknown extent. Alternatively, a wide enough spread of ${\mathrm{PL}}_{\mathrm{W}}$ in the data (see the next paragraph) may allow for the intercept estimate as a robust proxy for ${\mathrm{P}}_{0}$ for each individual in the data.

Tolerance is a difficult trait to estimate as the slope of a reaction norm of performance on ${\mathrm{PL}}_{\mathrm{W}}$ because this requires multiple measures of the independent (${\mathrm{PL}}_{\mathrm{W}}$) and dependent variable ($\mathrm{P}$), either within the individual (which would require longitudinal data over time, see the "Targeting complete resistance" section below) or, in the context of animal breeding, across genetically related individuals, such as within daughter groups of artificial insemination sires. With regard to quantification, recall that the approximate standard error ($\mathrm{se}$) of an estimated linear regression coefficient ${\widehat{\mathrm{b}}}_{\mathrm{yx}}$ of $\mathrm{y}$ on $\mathrm{x}$ is $\mathrm{se}\left({\widehat{\mathrm{b}}}_{\mathrm{yx}}\right)\approx \sqrt{\frac{{\widehat{\upsigma }}_{\mathrm{y}}^{2}}{\mathrm{n }{\widehat{\upsigma }}_{\mathrm{x}}^{2}}- \frac{{\widehat{\mathrm{b}}}_{\mathrm{yx}}^{2}}{\mathrm{n}}}$. It follows that reliable estimates of tolerance reaction norm slopes (particularly shallow ones with small ${\mathrm{b}}^{2}$) would require many observations (large $\mathrm{n}$) with a sufficiently large variation in the independent variable (here ${\mathrm{PL}}_{\mathrm{W}}$). Lough et al. [56] assessed the impact of small variation in ${\mathrm{PL}}_{\mathrm{W}}$ on tolerance estimates from the data used in the above case study, also demonstrating the usefulness of recording performance at zero ${\mathrm{PL}}_{\mathrm{W}}$, or using information from repeated ${\mathrm{PL}}_{\mathrm{W}}$ measurements to overcome the effects of small variance in the independent variable, x, as shown in their subsequent studies [32].

Given these financial, logistical and statistical hurdles, the question arises whether it is justified to collect ${\mathrm{PL}}_{\mathrm{W}}$ measures at all. Few breeding programs to date include routine measures of ${\mathrm{PL}}_{\mathrm{W}}$. A common approach in pig, poultry, and fish breeding programs is to select for reduced disease or mortality rates, or for high production performance based on data recorded on close relatives of nucleus selection candidates that are grown in commercial conditions with natural PL (e.g. [57–59]). The latter approach is equivalent to black-box selection for improved resilience with unknown ${\mathrm{PL}}_{\mathrm{E}}$ and ${\mathrm{PL}}_{\mathrm{W}}$. Mulder and Rashidi [14] performed simulations to compare such an approach to explicit selection for the component traits with ${\mathrm{PL}}_{\mathrm{W}}$ records, essentially by considering selection on an estimated breeding value (EBV) for resilience as index selection for resistance and tolerance. They concluded that such black-box selection for resilience is "an effective way to increase tolerance and resistance […] provided that both are not strongly unfavorably correlated". Based on their Fig. 3a, "strongly unfavorably correlated" starts at a genetic correlation of − 0.4; around that value, the product of the simulated $\mathrm{\Delta G}$ values of their resistance and tolerance traits (i.e. the $\mathrm{\Delta G}$ of resilience) approaches zero or even changes sign to become unfavorable. Note that their resistance and tolerance traits are equivalent to PL_W_ and β of our Fig. 1, and they calculate resilience as ${\upbeta \times \mathrm{PL}}_{\mathrm{W}}$, so that the signs are opposite to what we show here. In other words, as always when selecting on a composite trait, responses of the component traits are uncertain and depend on the genetic architecture around them: "the genetic correlation […] has a high impact on the selection responses in resistance and tolerance, and selection on resilience may lead to an unfavorable response in resistance or tolerance" [14]. Some practical consequences of ignoring the two component traits of disease resilience are considered in the next section.

#### Trade-offs between component traits

Antagonistic genetic correlations among traits under selection can represent trade-offs between various biological functions and can form important constraints in animal and plant evolution, and in livestock and crop breeding [4, 60 and chapter 30 in [61]]. The caveat of such trade-offs for disease resilience was pointed out by Råberg et al. [62]: "in the agricultural sector, attempts to select for increased yield in the face of parasite challenge may come to nothing (or even make things worse) if there is a trade-off between resistance and tolerance". Theoretical models predict that trade-offs between tolerance and resistance exist if these are controlled by alternative immune pathways that require energy resources [63–65]. If energy sources are limited (e.g. due to infection-induced anorexia or poor energy intake or maintenance), preferential resource allocation towards one set of mechanisms confers fitness costs to the other, with resulting trade-offs.

In livestock breeding, one-sided selection for increased production performance has been shown to lead to compromised animal robustness due to trade-offs between performance and fitness functions [66–68]. The reaction norm model studies for heat resilience mentioned in the previous section report nine estimates of the genetic correlation between performance and heat resilience; one of these is favorable (${\mathrm{r}}_{\mathrm{G}}$ = + 0.3), the other eight are unfavorable and range from –0.4 to –0.8.

Ignoring potential trade-offs between production and fitness traits in animal breeding programs can have strongly damaging consequences in terms of societal acceptability and commercial credibility (see [69] and [70], for a real-life example on litter size versus pre-weaning mortality in pigs). Sustainable breeding seeks to avoid this by including all the relevant component traits of a composite in the selection criterion. As outlined above, to date, this has not been achieved in the case of disease resilience.

The economic value of PRRS resilience traits in the above case study derives from a comparison of (i) responses to selecting for performance in an infection-free environment (${\mathrm{P}}_{0}$) with (ii) responses to selecting on $\mathrm{R}$ and $\mathrm{T}$ in an infectious environment, assuming that these three traits are genetically independent, thus ignoring potential trade-offs. A more appropriate selection criterion would be an index of EBV for ${\mathrm{P}}_{0}$, $\mathrm{R}$, and $\mathrm{T}$ to represent the breeding goal trait ${\mathrm{P}}_{\mathrm{PLE}}$, weighted by their MEV, as derived using Eqs. (2b) and (3b). Genetic improvement in the traits is commonly predicted (to support cost–benefit analyses) by selection index theory, which requires estimates of the genetic and phenotypic (co)variances of the traits. Lough et al. [32] provide several of these for the case of PRRS, but was not able to estimate the covariance between $\mathrm{R}$ and $\mathrm{T}$. Some of the other parameters can be derived as described in Additional file 2, with as the most striking result an approximate estimate of the genetic correlation between ${\mathrm{P}}_{0}$ and the tolerance slope $\upbeta$ of –0.99. Although these estimates need to be verified with better data, it is clear that it would be very difficult to generate $\mathrm{\Delta G}$ for growth potential and disease tolerance simultaneously in such a strongly antagonistic system.

To date, much of the R&D on disease resilience in livestock breeding has been on resistance. Considering tolerance as an explicit selection trait is a relatively novel idea (see e.g. [12] for reviews and recent exploratory examples, as well as [71] or [72]). If the genetic correlation between resistance and tolerance is antagonistic, a breeding program that selects for improved host resistance faces the risk of gradually decreasing host tolerance (and thus potentially also decreasing host resilience). These consequences are further exacerbated if the pathogen co-evolves successfully to neutralize host resistance [65]. In this case, a scenario with selection for increased resistance under antagonistic genetic correlations will paradoxically lead to reduced tolerance, to a higher infection load, and to neutralized resistance, and therefore to reduced resilience. And, as far as those unfavorable genetic correlations are actually unknown, selection for increased resistance could easily lead any animal breeding program into the societal acceptability and commercial credibility issues mentioned above, which is to be avoided whenever possible. But this would require reliable estimates of genetic correlations between disease resistance and tolerance to be obtained, which is a notoriously demanding task in general (e.g. [73]), and the hard-to-measure character of tolerance (see the above "Resilience component traits" section) only adds to this.

**Fig. 2:**
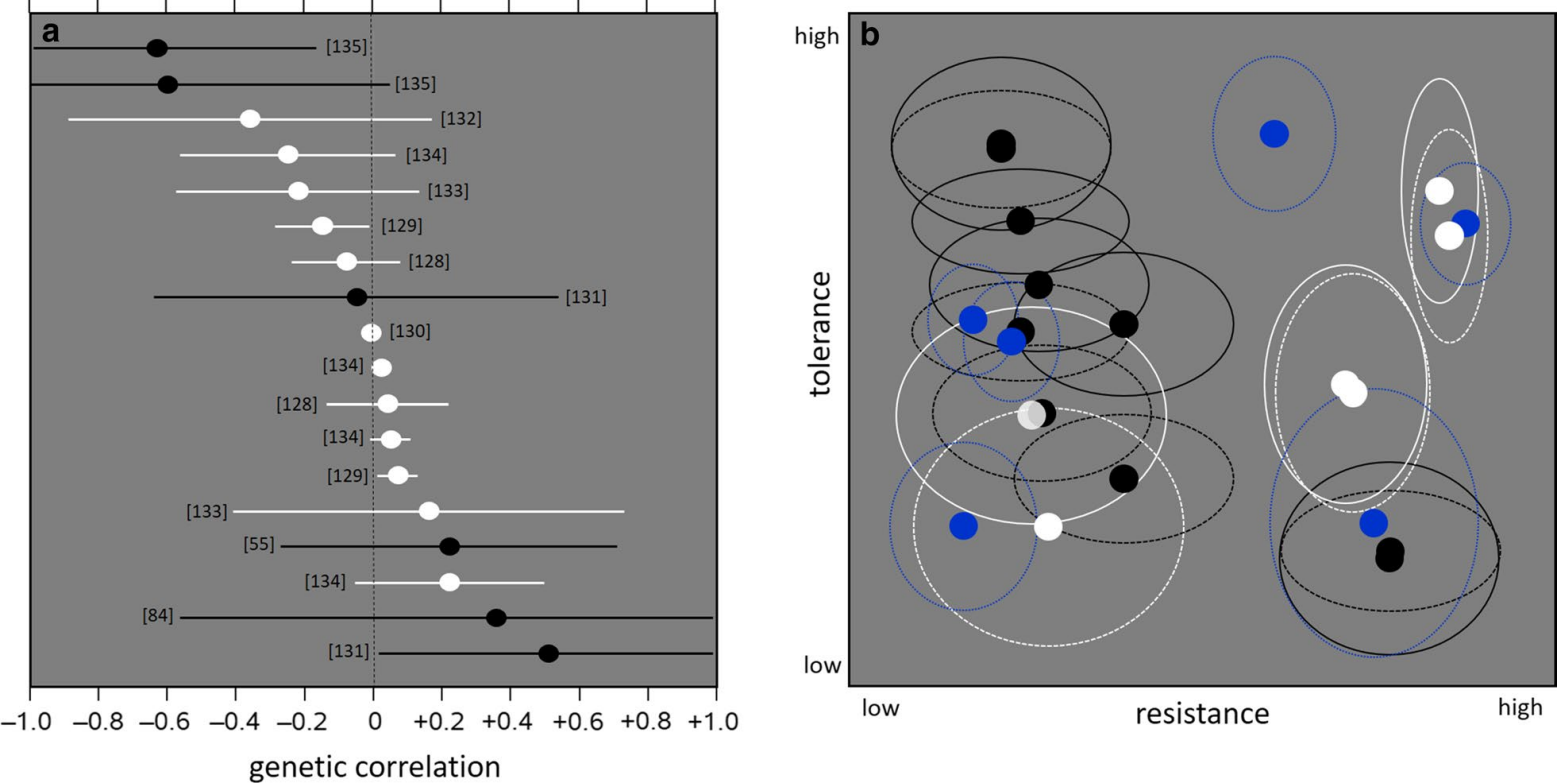
Published estimates of the relationship between resistance and tolerance in plants and animals. **a** Genetic correlation estimates between resistance and tolerance in various plant and animal species (quantified in ways that do not necessarily correspond to our "Theoretical framework" section of above). The error bars represent the 95% confidence interval (± 1.96 standard errors around the estimate, some of these were derived from the published P values). Black symbols: infectious diseases, white symbols: other stressors. Data from [128] (*Arabidopsis* versus insects), [129] (*Ipomoea* versus insects), [130] (*Brassica* versus frost), [131] (*Mimulus* versus mosaic virus), [55] (tiger shrimp versus Taura virus), [132] (chicken versus ascites), [133] (*Solanum* versus insects), [134] (*Arabidopsis* versus frost and heat), [135] (sheep versus nematode), [84] (turbot versus skin parasite). **b** Estimated means with 68% confidence ellipsoids (± 1 standard error around the bivariate mean) for tolerance and resistance of inbred mouse strains to three different types of pathogens. Black data points: tolerance of five inbred mouse strains to the malaria parasite *Plasmodium chabaudi* (regression of body weight [solid ellipsoids] or erythrocyte density [dashed] on pathogen load) in relation to the reverse of pathogen load (data from Fig. 3 in [22]; ~ 30 animals per subclass). White data points: tolerance of three inbred mouse strains to the nematode *Heligmosomoides bakeri* (correlation of carcass weight with two counts of pathogen load: solid and dashed ellipsoids) in relation to the reverse of pathogen load (data from Table 3 and Fig. 1 in [136]; 10 animals per subclass). Blue data points: tolerance of four inbred mouse strains to the bacterium *Listeria monocytogenes* (regression of scaled body weight on pathogen load) in relation to the reverse of pathogen load (data from Fig. 2 in [114]; 10 animals per strain, with two strains further subdivided into survivors and non-survivors)

This is illustrated in Fig. 2a, which depicts published estimates of the correlation between resistance and tolerance, along with their confidence intervals, from various studies in animals and plants. The correlation estimates span a wide range from − 0.6 to + 0.5, so in practice one could expect any value for the host and pathogen population under study. Without actual data analysis, it is impossible to predict whether trade-offs between resistance and tolerance exist. Furthermore, most of the confidence intervals include a zero correlation, demonstrating the high uncertainty in quantifying this relationship, even when data are available.

Differences in the approaches to quantify resistance and tolerance, with ${\mathrm{PL}}_{\mathrm{W}}$ only included in some approaches, may account for some of the uncertainty in the correlation estimates and their observed variation between the studies in Fig. 2a. Results from more unified experimental approaches are shown in Fig. 2b, which illustrates the relationship between resistance and tolerance in various inbred mouse strains against (i) malaria, (ii) a nematode, or (iii) a bacterium, where resistance was estimated as the inverse of ${\mathrm{PL}}_{\mathrm{W}}$ and tolerance as the regression of a performance trait on ${\mathrm{PL}}_{\mathrm{W}}$, as outlined in the above "Theoretical framework" section. Similar to the correlation estimates for outbred livestock (Fig. 2a), the relationship between resistance and tolerance varies considerably between types of host and between pathogen strains.

In summary, evidence suggests that the genetic correlation between resistance and tolerance can be very favorable or very unfavorable or anything in between, and is expensive to quantify; not only because genetic correlation estimates require large datasets in general, but also because it requires recording of $\mathrm{PL}$, which is expensive in its own right. Thus, although conceptually attractive and analytically elegant, the reaction norm approach constitutes an expensive model for routine estimation of resilience breeding value. The next section discusses potential routes forward.

### The future: four alternative approaches

#### The purist approach

The "purist" approach for the setup of a disease resilience breeding program would be to start with $\mathrm{PL}$ (and production) recording until the data volume is sufficient for estimating the genetic correlation between resistance and tolerance, estimate the genetic parameters, and evaluate the possibly antagonistic system. If trade-offs exist, continued routine recording of $\mathrm{PL}$ would be required for decomposing resilience into its component traits to be implemented into a selection index that effectively balances the trade-offs. However, when there is no evidence for such trade-offs, decomposing resilience is not necessary and reaction norms can be calculated based on cheaper proxies for the environmental PL, such as the mean production performance of each individual's contemporary group (as in Mulder and Rashidi's [14] simulated scenario without $\mathrm{PL}$ recording, mentioned in the above "Resilience component traits" section). This approach is commonly used to model general resilience to a nondescript mixture of infectious and non-infectious stressors (e.g. [54]). Real-life studies using such a model were summarized in Table 3 of Knap and Su [74], who also showed that the statistical method of Su et al. [75] to estimate all parameters of the reaction norm system (i.e. ${\mathrm{P}}_{0}$, $\upbeta$, and the value of the independent variable) in a single-step analysis produces more robust statistics than the conventional contemporary group approach. This method requires production records only, but poses strong demands on their volume and genetic structure; making use of a genomic relationship matrix in the mixed model equations for this method is expected to increase the accuracy of all the estimates, including those of the independent variable (i.e. the proxy for PL_E_).

One of the major obstacles of this purist approach to a resilience breeding program that appropriately handles trade-offs is the necessity to measure $\mathrm{PL}$, at least initially. For systems for which this is unfeasible, various approaches have been proposed to use proxy traits to quantify host resistance or the infection challenge prevalent in a specific environment, e.g. immune parameters or routinely collected health records (e.g. [76–80]). These proxy traits would need to be carefully evaluated before implementing them into a breeding program, which requires significant investment in time and money.

With the advent of high-throughput genomics and high-resolution automated phenotyping technologies, alternative solutions to breeding disease resilient livestock that do not use classical reaction norms are now emerging. These alternatives are considered in the next sections.

#### Targeting beneficial QTL for both resistance and tolerance

Evidence from a plethora of genome-wide association studies (GWAS) in livestock suggests that disease resistance, tolerance, and resilience are mostly under polygenic control, with a handful of genes of relatively large effect (e.g. [7, 21, 81–83]). This implies that genetic improvement of resilience traits will be necessarily gradual and may require many generations of selection to achieve complete resistance or tolerance, if at all possible. One possible approach to minimize the risk of undesirable outcomes of selection due to hidden trade-offs may be to focus on genomic loci with identified large positive effects on both resistance and tolerance (e.g. [84]). Alternatively, recalling the challenges of estimating genetic parameters for tolerance reaction norms, one could focus on candidate loci with known large effects on disease resistance and then determine their effects on tolerance. This has been exemplified for PRRS, where GWAS identified a region on pig chromosome 4 with a major QTL that explained 10 to 20% of the genetic variance for resistance and resilience to PRRS [85]. Subsequent studies found that this QTL is also significantly associated with tolerance to PRRS, with the genetically more resistant pigs being also more tolerant [32]. Selection on such QTL with known beneficial pleiotropic effects on both traits may be a promising short-term strategy to gradually improve resistance and tolerance simultaneously in the absence of reliable genetic correlation estimates.

#### Targeting complete resistance or complete tolerance

One way to avoid undesirable scenarios of unknown genetic correlations (see the above "Trade-offs between component traits" section) would be to increase resistance or tolerance (both continuous traits, as per above) not gradually but completely, preferably in a few rapid modification steps. Eqs. (1a) and (1b) and Fig. 1 illustrate that completely resistant animals do not need any tolerance to achieve high performance levels under infectious challenge; and likewise, for completely tolerant animals the pathogen load level is irrelevant.

Such rapid modification steps would require a host–pathogen mechanism with a high host heritability and a high prediction accuracy for the trait under consideration, such that selection would achieve results quickly. In practice, this is most easily achieved if the trait has a simple genetic architecture, i.e. if it is to a large extent controlled by a single gene. Here, we give three examples where such rapid improvements were achieved through selection on a single DNA marker, in two cases without significant knowledge of the underlying biological mechanisms.

First, marker-assisted selection for resistance to transmissible spongiform encephalopathy (scrapie) in sheep, which is based on selection for particular genotypes of the *PNRP* gene that modulates much of the variation in susceptibility to developing scrapie. In the UK, an increase in the frequency of the favorable allele in young rams from 50 to 69% (see Table 2 in [86]) reduced the observed national scrapie prevalence to close to zero in 9 years i.e. in three to four generations (see Table 14 in [87]). In the Netherlands, an increase of the frequency of the favorable allele in the ewe population from 38 to 65% caused a similar prevalence reduction in 7 years, i.e. in three generations (see Fig. 5 in [88]).

Second, marker-assisted selection in pigs for resistance against *Escherichia coli* caused by a lack of the cell receptors for the attachment of the bacterial adhesins [89]. *E. coli* F4 resistance in the sow populations of two Danish breeds increased from 0 to 80%, and from 5 to 100%, in 6 years, i.e. in four generations (see Fig. 11 in [5]). *E. coli* F18 resistance in the artificial insemination boar populations of two Swiss breeds increased from 39 to 100% in 4 years, and from 30 to 100% in 7 years, i.e. in three to five generations [90].

Third, marker-assisted selection for reduced mortality due to infectious pancreatic necrosis (IPN) in Atlantic salmon, based on a DNA marker developed by Moen et al. [91] and Houston et al. [92], which explains ~ 98% of the genetic variation in mortality. Selection for this gene reduced the annual number of IPN outbreaks in Norwegian farms from 223 to 23 in 9 years, i.e. in three salmon generations [93], tinyurl.com/y9gq3wyc). In light of the trade-off concerns raised in the above "Obstacles for genetic improvement" section, the outcome of this black-box selection for resilience is remarkable because the impact of the underlying gene on resistance and tolerance was unknown. Only later studies revealed that the favorable allele for reduced IPN mortality conferred increased resistance to infection as well as to infection transmission (thus effectively reducing ${\mathrm{PL}}_{\mathrm{E}}$ in the population), without significantly impacting the mortality rate of infected individuals, i.e. their tolerance to infection [16]. In other words, the observed increase in resilience was due to a fortunate combination of favorable or neutral gene effects on the various resilience components simultaneously. There are currently too few real-world examples of marker-assisted selection for improved disease resistance or resilience to determine whether this combination is the norm or the exception. However, the examples shown in Fig. 2 would suggest that such good fortune should not be taken for granted.

An alternative route to achieve complete resistance (or tolerance) would be to move away from the classical gradual selection approaches and exploit (i) detailed biological knowledge of the relevant resilience mechanism, and (ii) novel genomic technology to manipulate it. Genome editing is a promising powerful methodology, and we can expect much development in this field for animal breeding, just as in plant breeding (e.g. [94] for resistance against mildew in wheat). Ruan et al. [95] and Proudfoot and Burkard [96] describe the current state of the art of genome editing in animal breeding. PRRS resistance in pigs constitutes one of the most promising examples, where a disruption of the *CD163* gene (either through knock-out of the complete gene or a simple deletion of its exon 7) makes pigs completely resistant to infection with the PRRS virus, in the latter case without loss of the original physiological functionality of the protein [97–100].

An important point here is that this approach could not have been developed based on scanning procedures such as GWAS that rely on natural polymorphisms to detect useful information. In earlier in-vitro studies, the *CD163* gene was shown to code for the host protein domain that is hijacked by the virus to enable the release of its genome into the host macrophage [101]. However, extensive GWAS and *CD163*-focused sequencing studies have detected no natural polymorphism at the relevant locus [102, 103], or produced signals in very different parts of the gene [104, 105].

In summary, achieving complete genetic resistance or tolerance to infection requires identification of genes with a very large effect on these traits. The few existing examples in livestock suggest that such genes are rare or difficult to identify without detailed biological knowledge of the relevant resilience mechanisms. Novel genomic tools may accelerate the discovery of such genes. The main logistical challenge will be to rapidly disseminate the beneficial alleles into the production pyramid, before the pathogen evolves to neutralize the desired gene function.

#### Moving towards dynamic resilience indicators

A fourth alternative for genetic improvement of disease resilience is to temporarily step away from the reaction norm approach to quantify resilience, and consider fundamentally different approaches that are emerging from resilience studies in other fields of research. Such alternative approaches, with origins in mathematical dynamical systems theory, have recently been put forward for livestock breeding, where resilience is defined as "the capacity of the animal to be minimally affected by disturbances or to rapidly return to the state pertained before exposure to a disturbance" [28, 29, 106]. Thus, resilience in this context characterizes how an animal responds, over time, to disturbances such as infection.

To estimate the capacity of an animal to resist or to rapidly recover from the perturbation caused by this disturbance, longitudinal measures of performance and possibly health before, during, and ideally after the perturbation period are required. Such time-series data of relevant response variables are increasingly becoming routinely available in livestock systems through the rapid rise in technologies for automated data recording of traits such as milk yield, body weight, and feed intake, as well as indicators of infection severity, immune responsiveness, or health, such as somatic cell counts or infrared measures of body temperature. We refer to [107, 108] for a comprehensive review of recent advances in biosensors and wearable technologies and associated data analytics that allow real-time monitoring of the physical and health state of animals. The data generated by such precision livestock farming technologies offer new opportunities to explore new analytical tools to generate dynamic resilience indicators [109]. Below we discuss some recent advances relevant to livestock breeding.

### Two-dimensional resilience trajectories

We come back to the PRRS data introduced at the end of the above "Case study" section, to illustrate how individual time-series data of ${\mathrm{PL}}_{\mathrm{W}}$ and performance can give rise to informative two-dimensional (2D) resilience trajectories. A pig's static resilience to PRRS was described there by its average growth rate over a fixed infection period, where static resistance was quantified by the area under the viremia curve over that period, and (static) tolerance was estimated as the slope of the reaction norm of growth rate against AUC(logVL); both traits showed significant genetic variation in that study [56].

**Fig. 3:**
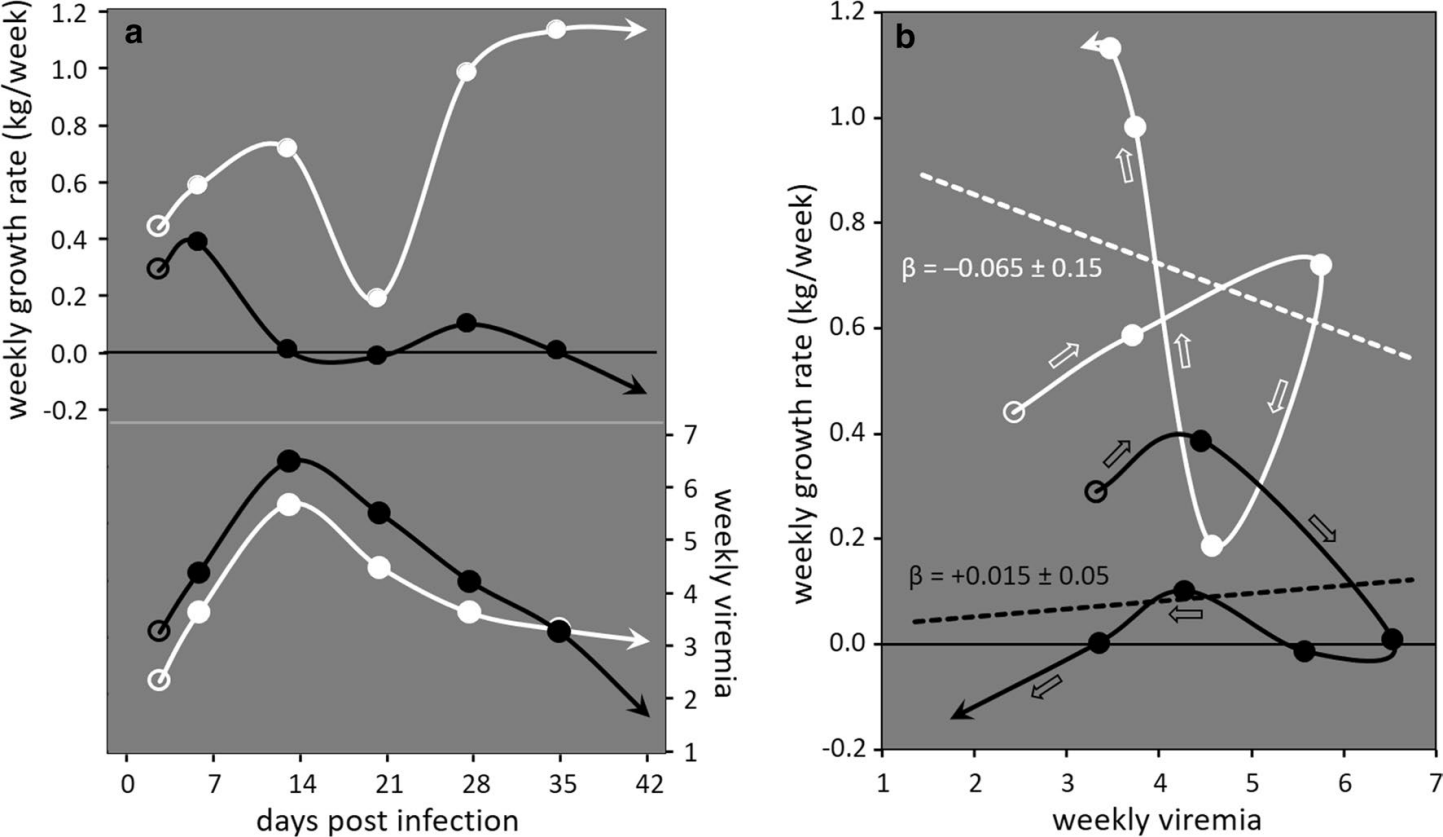
Reaction norms and dynamic resilience trajectories constructed from longitudinal measures of pathogen load and performance. **a** Temporal profiles for growth rate (${\mathrm{P}}_{\mathrm{PLW}}$) and viremia (${\mathrm{PL}}_{\mathrm{W}}$) of two pigs infected with the PRRS virus. **b** The associated reaction norms and dynamic resilience trajectories. Data from [32]. Datapoints represent observations (the earliest ones as open symbols, the final ones as arrowheads), the solid trendlines in (**b**) show time trends. The dashed lines in (**b**) represent the linear regression through the data (i.e. the reaction norm) with slope estimate β and its standard error

Figure 3 shows data on two animals from that same challenge experiment: the courses of ${\mathrm{PL}}_{\mathrm{W}}$ and growth rate over time (Fig. 3a) and, more informatively, the associated reaction norms and the dynamic trajectories of weekly growth rate of each individual plotted against its weekly ${\mathrm{PL}}_{\mathrm{W}}$, i.e. AUC(logVL) (Fig. 3b). Both reaction norms have slope estimates with high standard errors, such that neither differs significantly from zero or from the other one (P > 0.67). The dynamic resilience trajectories in Fig. 3b are constructed using the same traits (growth rate and ${\mathrm{PL}}_{\mathrm{W}}$) as the static resilience measures, but harness the coupled time trends in these traits. In this manner, the trajectories not only illustrate how an animal’s weekly change in growth rate is associated with change in infection severity at that time, but also the route to recovery to its pre-infection performance state corresponding to zero ${\mathrm{PL}}_{\mathrm{W}}$. In this example, both animals show similar viremia trends (i.e. similar resistance), but the black pig does not grow over the whole 7-week infection period, whereas the white pig experiences a temporary reduction in growth associated with the initial decline in viremia, followed by a steady increase in growth as ${\mathrm{PL}}_{\mathrm{W}}$ reduces to zero. Overall, the 2D dynamic trajectories in Fig. 3b reveal that the black pig is less resilient to the infection than the white pig, although the slope estimates, which do not take the time course of the measurements into account, would suggest the opposite. Thus, this example highlights the potential importance of capturing the dynamic aspects of disease resilience.

More generally, such 2D resilience trajectories, which can also be constructed using different resistance or performance indicators (e.g. immune response measures [110]), provide relevant temporal information on animal’s responses that cannot be captured by static reaction norms. For example, they reveal which stages of infection are associated with the strongest loss of performance, which may provide useful insight into underlying resilience mechanisms and target genes, or for devising effective timely and targeted treatment [111]. Dynamic trajectories also have the advantage over reaction norms that they capture how resistance and tolerance mechanisms interact over time, without the need to disentangle and explicitly estimate these traits [56, 112].

In spite of these apparent advantages, implementation of such dynamic trajectories into practical breeding programs will require novel analytical approaches to derive and validate meaningful and reliable indicators that capture the trajectory characteristics and that lend themselves to routine genetic evaluation. Methods for analysing such complex trajectories, the patterns of which cannot be described by mathematical functions, have just started to emerge [110, 113–116], and as such the value to animal breeding is yet to be determined.

### Black-box one-dimensional dynamic resilience indicators

When longitudinal measures of ${\mathrm{PL}}_{\mathrm{W}}$ or other immune parameters are not available for constructing 2D resilience trajectories, dynamic resilience indicators of individual animals can be constructed from temporal profiles of production or immune measures alone, such as the growth rate profile in Fig. 3a. This has been exemplified in recent studies that derived such indicators from temporary reductions or day-to-day variations in milk yield or body weight [117, 118], or from daily feed intake data [28, 106, 119] or natural antibody titres [120]. Although these studies differ in their exact approaches to derive dynamic resilience indicators, the common underlying assumption is that a single or several (known or unknown) stressors cause temporal deviations in the performance traits, and that resilience can be quantified by the scale, pattern, or duration of these deviations: more resilient animals experience less pronounced deviations from their target performance trajectory [106, 109].

In contrast to the above 2D resilience trajectories that describe how infection severity and performance interact over time, the one-dimensional (1D) dynamic resilience indicators that are solely based on deviations in performance or any other characteristic that can be easily monitored over time, must be assessed with regards to their informative value for resilience [106]. For example, anorexia (a temporary reduction of food intake) forms an important coping mechanism of infected animals [121]. Thus, a large deviation in feed intake may correspond to high rather than low resilience. Putz et al. [119] found moderate to strong genetic correlations of (i) disease resilience indicators based on daily variability in feed intake or feed intake duration with (ii) mortality and treatment rate in a natural disease challenge environment comprising a cocktail of pathogens. Later studies conducted in the same polymicrobial challenge facilities identified natural antibody titres circulating in healthy pigs as potential indicators for disease resilience that were also genetically correlated with fluctuations in feed intake [120]. Similarly, Elgersma et al. [118] found that on a genetic level, cows with low variance in milk yield deviations over time tended to have fewer production-related diseases and longer longevity. By contrast, Berghof et al. [117] found close-to-zero genetic correlations between body weight deviations and natural antibodies, questioning the validity of the former as disease resilience indicators in their dataset.

## Pros and cons of dynamic resilience indicators

Similar to reaction norms calculated with or without $\mathrm{PL}$ measures, 2D resilience trajectories based on measures of $\mathrm{PL}$ and performance are more informative for a specific disease than 1D trajectories based on performance records alone. Capturing more information generally leads to increased understanding of the biological system and potential trade-offs (e.g. the relationship between resistance and tolerance) and hence also to greater chances of bringing it under control [122]. However, these advantages need to be weighed against the additional recording requirements in a cost–benefit analysis.

Black-box 1D dynamic resilience indicators may offer attractive resilience measures in situations where the stressor or ${\mathrm{PL}}_{\mathrm{W}}$ are unknown, or where multiple pathogens and other stressors are likely to be present simultaneously, such as in a typical production farm [29, 106, 119]. Hence, they have been coined as "general resilience" in contrast to resilience to a specific disease [119]. However, this terminology may be somewhat misleading as it still applies to one particular environment only. It is not known whether an animal with high resilience in one environment would also have high resilience in an environment where different combinations of pathogens are circulating but it is unlikely that similar resilience responses will occur across environments with different pathogen load. This implies that across farms (or over time) the variable ${\mathrm{PL}}_{\mathrm{E}}$ levels (or relevant proxies as described above) will still have to be quantified. Longitudinal performance records on individuals or (more realistically) groups of relatives in a range of environments could then produce resilience estimates both within and across environments, using the dynamic resilience indicators within each environment as the response variable of the across-environmental reaction norm model. In other words, dynamic resilience indicators complement but do not replace the reaction norm model; the latter remains the only method to accurately estimate resilience across environments.

## Final remarks

All of the above has focused on resilience of individual animals. At a higher integration level, herd resilience depends on "the adaptive capacity of the animals in the herd, together with the management decisions that affect the performance trajectories and local environment of the animals" [123]. In terms of disease resilience, a crucial component of this "local environment of the animals" is the environmental pathogen load, which is often largely influenced by pathogen transmission among animals in the herd. This introduces the animal-intrinsic trait host infectivity, i.e. the propensity of an animal, once infected, to transmit infection to others [124]. Host infectivity, resistance, and tolerance are likely interdependent, as the capacity of an animal to infect its groupmates logically depends on its own resistance and tolerance to the pathogen. In particular, tolerant animals that do not eradicate the pathogen from their system via resistance, may continue shedding it into their micro-environment [15, 125, 126]. Thus, individual resilience, when dominated by tolerance, may be counterproductive to herd resilience. It follows that a complete quantitative treatment of the topic of disease resilience should include the trait host infectivity, in addition to resistance and tolerance; see e.g.[15, 16], and [127] for approaches to calculate the economic value of selective breeding for reduced disease transmission. Similarly, future studies should also consider the impact of breeding for disease resilience and its component traits on pathogen evolution. As pointed out in the above "Trade-offs between component traits" section, pathogen evolution could easily neutralize genetic gains in disease resilience. However, these aspects are beyond the scope of this paper.

### Conclusions

Reaction norm models have proven useful for quantifying the relative effects on disease resilience of environmental pathogen load and of the animal's production potential, resistance and tolerance, and for quantifying the so far unknown economic values. Their effective implementation in livestock breeding programmes is currently hampered by the lack of adequate tools to measure, or accurately estimate, these resilience component traits, in particular pathogen load. Likely trade-offs between resilience component traits, if not properly accounted for, can jeopardize genetic improvement of disease resilience. Recent advances in affordable and accurate high-throughput genomic and high-resolution automated phenotyping technologies, as well as in genome editing, accompanied by promising developments in statistical methods adapted to these data, offer exciting new opportunities to overcome these shortcomings and breed livestock with greater genetic resilience to current and future infectious diseases. Such data and methods will enable construction of informative dynamic resilience indicators to optimize animals' responses to specific pathogen challenges, and reaction-norm models to identify animals with high genetic resilience to a wide range of diseases, should such generic resilience exist.

## Supplementary information

**Additional file 1: Table S1.** Estimated cost of combating infectious livestock diseases on the national level. Additional information about Table 1 of the main text [137–159].

**Additional file 2:** Derivation of correlation estimates for performance potential and tolerance for the PRRS case study. Mathematical derivations and results table.

## Data Availability

This article uses only data already published. We refer to the original sources for information on data availability.
