# Supplementary material for: Why breed disease-resilient livestock, and how?
Source: Genet Sel Evol. 2020 Oct 14;52:60. doi: 10.1186/s12711-020-00580-4 (PMC7557066; doi:10.1186/s12711-020-00580-4)
Supplement: Supplementary file 1 — Additional file 1: Table S1. Estimated cost of combating infectious livestock diseases on the national level. Additional information about Table 1 of the main text [137–159]. [file 12711_2020_580_MOESM1_ESM.docx]

**Additional File 1.**

**Table S1. Cost of fighting disease versus the value of genetic improvement in (re)production traits.**

| area | year | species | disease^a^ | feature | currency | total cost (M / year) | herd size^b^ (M head) | cost per head | ΔG per head | cost / ΔG |
| --- | --- | --- | --- | --- | --- | --- | --- | --- | --- | --- |
| Netherlands | 1997 | pig | CSF | cost of outbreak control^c^ | EUR | 2,340^d^ | 15 | 153 |  |  |
| Taiwan | 1997 | ungulates | FMD | direct cost + export losses^c^ | USD | 1,600^d^ | 12 | 135 |  |  |
| UK | 2001 | ungulates | FMD | cost of outbreak control^c^ | GBP | 8,000^d^ | 42^be^ | 190 |  |  |
| South Korea | 2010 | ungulates | FMD | cost of outbreak control^c^ | USD | 1,856^f^ | 16 | 115 |  |  |
| China | 2019 | pig | ASF | cost of outbreak control^c^ | USD | 25,500^g^ | 236^g^ | 108^g^ |  |  |
| Australia | 2006 | sheep | parasites | production losses + control cost | AUD | 649^h^ | 105 | 6.2 | 0.81^i^ | 7.6 |
| Netherlands | 2007 | ruminants | Bluetongue | cost of outbreak control | EUR | 170^j^ | 5.2^bk^ | 37.3 | 14^m^ | 2.7 |
| England | 2010 | cattle | BTB | routine control cost | GBP | 109^n^ | 5.5^e^ | 19.9 | 3.8^ko^ | 5.2 |
| Canada | 2010 | pig | PRRS | production losses | CAD | 130^p^ | 21 | 6.1 | 1.2^q^ | 5.8 |
| USA | 2013 | pig | PRRS | production losses | USD | 1,142^r^ | 112 | 10.2 | 2.2^s^ | 4.7 |
| Europe^t^ | 2013 | pig | PRRS | production losses | EUR | 1,660^u^ | 252 | 6.6 | 1.6^v^ | 4.1 |

The table shows the estimated cost of fighting infectious livestock diseases on the national level, compared to the value of estimated genetic trend in production and reproduction traits during a five- to eight-year period around the reporting year (ΔG, in money per head per year).

a: ASF: African swine fever; BTB: bovine tuberculosis; CSF: classical swine fever; FMD: foot and mouth disease; PRRS: porcine reproductive and respiratory syndrome.

b: Approximate total numbers of animals (mature and growing) alive in that calendar year, mainly from [www.fao.org/faostat/](http://www.fao.org/faostat/).

c: Very high cost due to massive culling.

d: [137], table 7.

e: [138]

f: [139]

g: 236M pigs were culled in 2019 [140] (p. 3); the cost was set at CNY 1200-1500 per sow and 600-800 per growing pig [141, 142], weighted here as 1:9.

h: [143]

i: [144], appendix 4.2.

j: [145]

k: [146]

m: Trend for Holstein cattle: [147]

n: [148]

o: [149]

p: [150]

q: [151], appendices 5 and 8.

r: [152]

s: Trend reported for PIC, with ~45 % USA market share at that time: [153], p. 17.

t: Austria, Belgium, Denmark, France, Germany, Italy, Netherlands, Poland, Russia, Spain and UK.

u: [154].

v: Mean value of trends reported for Denmark ([155], table 2 p. 11; [156], p. 16), Netherlands ([157]; pers.comm. R. Hovenier, 2018), Germany ([158]; pers.comm. J. Dodenhoff, 2018) and France [159].
