# Supplementary material for: Why breed disease-resilient livestock, and how?
Source: Genet Sel Evol. 2020 Oct 14;52:60. doi: 10.1186/s12711-020-00580-4 (PMC7557066; doi:10.1186/s12711-020-00580-4)
Supplement: Supplementary file 2 — Additional file 2: Derivation of correlation estimates for performance potential and tolerance for the PRRS case study. Mathematical derivations and results table. [file 12711_2020_580_MOESM2_ESM.docx]

**Additional file 2**

**Derivation of correlation estimates for performance potential and tolerance for the PRRS case study**

The variance of the performance potential, var(P_0_), can be derived from the variance of realized performance, var(P_PBE_), as follows.

P_PBE_ = P_0_ + β × PB_W_ =

$$= {(P}_{0}+\beta\bar{\mathrm{PB}_{W}} )+\beta\left( \mathrm{PB}_{W}-\bar{\mathrm{PB}_{W}} \right)$$

…where β is the (negative) tolerance slope, $\bar{\mathrm{PB}_{W}}$ is the mean PB_W_ in the data, and
${(P}_{0}+\beta\bar{\mathrm{PB}_{W}} )$ is $P_{\bar{\mathrm{PBW}}}$ : the performance level at $\bar{\mathrm{PB}_{W}}$.

Now,

$${cov(P}_{0}+\beta\bar{\mathrm{PB}_{W}} , \beta)=cov\left( P_{0}, \beta\right)+\bar{\mathrm{PB}_{W}} var(\beta)$$

$\Rightarrow cov\left( P_{0}, \beta\right)={cov(P}_{0}+\beta\bar{\mathrm{PB}_{W}} , \beta)-\bar{\mathrm{PB}_{W}}\mathrm{var}\left( \beta\right)=$

$=cov(P_{\bar{\mathrm{PBW}}} , \beta)-\bar{\mathrm{PB}_{W}}\mathrm{var}\left( \beta\right)$ (A2-1)

Similarly,

$${var(P}_{0}+\beta\bar{\mathrm{PB}_{W}} )=var\left( P_{0} \right)+\left( \bar{\mathrm{PB}_{W}} \right)^{2} var(\beta)+2\bar{\mathrm{PB}_{W}}\mathrm{cov}\left( P_{0}, \beta\right)$$

$$\Rightarrow var\left( P_{0} \right)={var(P}_{0}+\beta\bar{\mathrm{PB}_{W}} )-\left( \bar{\mathrm{PB}_{W}} \right)^{2}\mathrm{var}\left( \beta\right)-2\bar{\mathrm{PB}_{W}}\mathrm{cov}\left( P_{0}, \beta\right)$$

Substituting from equation (A2-1) gives

$$\mathrm{var}\left( P_{0} \right)={var(P}_{0}+\beta\bar{\mathrm{PB}_{W}} )-\left( \bar{\mathrm{PB}_{W}} \right)^{2}\mathrm{var}\left( \beta\right)-2\bar{\mathrm{PB}_{W}} \left[ {cov(P}_{0}+\beta\bar{\mathrm{PB}_{W}} , \beta)-\bar{\mathrm{PB}_{W}} var(\beta) \right]=$$

$={var(P}_{\bar{\mathrm{PBW}}})+\left( \bar{\mathrm{PB}_{W}} \right)^{2}\mathrm{var}\left( \beta\right)-2\bar{\mathrm{PB}_{W}} {cov(P}_{0}+\beta\bar{\mathrm{PB}_{W}} , \beta)=$

$={var(P}_{\bar{\mathrm{PBW}}})+\left( \bar{\mathrm{PB}_{W}} \right)^{2}\mathrm{var}\left( \beta\right)-2\bar{\mathrm{PB}_{W}} cov(P_{\bar{\mathrm{PBW}}}, \beta)$ (A2-2)

Table A2-1 lists the estimated values for these parameters as they were provided in [32] or follow from their data, and the resulting values for var(P­_0_), cov(P_0_, β) and r(P_0_, β) as they follow from equations (A2-1) and (A2-2). The covariance between P_0_ and T can be approximated from cov(P_0_, β) assuming normality and using Stein's lemma (tinyurl.com/yc3455qc): ${cov(P}_{0},T)\approx\bar{\beta^{-2}} \times cov\left( P_{0}, \beta\right)$. But for a *genetic* correlation this requires the individual breeding values of β, and we do not have these available.

**Table A2-1. Parameter estimates for and from equations (A2-1) and (A2-2). Except for** $\bar{\mathbf{PB}_{\mathbf{W}}}$**, all entries are on the genetic level.**

| parameter | value |
| --- | --- |
| $\bar{\mathrm{PB}_{W}}$ | 117.5 |
| ${var(P}_{\bar{\mathrm{PBW}}})$ | 2.900 × 10^–6^ |
| var(β) | 8.530 × 10^–9^ |
| $cov(P_{\bar{\mathrm{PBW}}}, \beta)$ | 1.020 × 10^–10^ |
| var(P_0_) | 1.206 × 10^–4^ |
| cov(P_0_, β) | –1.002 × 10^–6^ |
| r(P_0_, β) | –0.99 |
